# Aurora-A promotes the establishment of spindle assembly checkpoint by priming the Haspin-Aurora-B feedback loop in late G2 phase

**DOI:** 10.1038/celldisc.2016.49

**Published:** 2017-01-10

**Authors:** Fazhi Yu, Ya Jiang, Lucy Lu, Mimi Cao, Yulong Qiao, Xing Liu, Dan Liu, Terry Van Dyke, Fangwei Wang, Xuebiao Yao, Jing Guo, Zhenye Yang

**Affiliations:** 1 Key Laboratory of Innate Immunity and Chronic Disease of CAS, Innovation Center for Cell Biology, School of Life Sciences, Hefei National Laboratory for Physical Sciences at Microscale, University of Science and Technology of China, Hefei, China; 2 Center for Advanced Preclinical Research, the Center for Cancer Research, National Cancer Institute, Frederick, MD, USA; 3 Anhui Key laboratory for Cellular Dynamics & Chemical Biology, Chinese Academy of Sciences Center for Excellence in Molecular Cell Science, Hefei, China; 4 Life Sciences Institute and Innovation Center for Cell Biology, Zhejiang University, Hangzhou, China

**Keywords:** Aurora-A, chromosomal passenger complex, H3T3-ph, Haspin, spindle assembly checkpoint

## Abstract

Aurora-A kinase functions mainly in centrosome maturation, separation and spindle formation. It has also been found to be amplified or overexpressed in a range of solid tumors, which is linked with tumor progression and poor prognosis. Importantly, Aurora-A inhibitors are being studied in a number of ongoing clinical trials. However, whether and how Aurora-A has a role in the regulation of the mitotic checkpoint is controversial. Additionally, the function of nuclear-accumulated Aurora-A in late G2 phase is not clear. Here we show that knockout, inhibition or blockade of the nuclear entry of Aurora-A severely decreased the centromere localization of Aurora-B and the phosphorylation of histone H3 threonine 3 (H3T3-ph) mediated by the kinase Haspin in late G2 phase. We further reveal that nuclear-accumulated Aurora-A phosphorylates Haspin at multiple sites at its N-terminus and that this promotes H3T3-ph and the rapid recruitment to the centromere of the chromosomal passenger complex. In addition, Aurora-A facilitates the association of Aurora-B with their common substrates: Haspin and Plk1. Notably, these functions of Aurora-A are mostly independent of Plk1. Thus we demonstrate that, in late G2 and prophase, Aurora-A phosphorylates Haspin to trigger the Haspin-H3T3-ph-Aurora-B positive feedback loop that supports the timely establishment of the chromosomal passenger complex and the mitotic checkpoint before spindle assembly.

## Introduction

Aurora kinases are conserved serine/threonine kinases. Although they share very similar catalytic kinase domains, Aurora-A and Aurora-B kinases have distinct functions that are related to their subcellular distributions [1–3]. Aurora-A is localized at centrosomes, spindle poles and the microtubules of the spindle distal end where it performs functions in centrosome maturation and separation prior to mitosis and in the subsequent bipolar spindle formation during mitosis. It is also required for efficient mitotic entry and chromosome alignment [1, 3, 4]. Amplification and overexpression of Aurora-A kinase has been found in various solid tumors and is regarded as a biomarker of a poor prognosis [1]. Thus Aurora-A is a rationale target for anticancer therapy, and Aurora-A inhibitors are being studied in a number of clinical trials. As the catalytic subunit of the chromosomal passenger complex (CPC), which also contains the inner centromere protein INCENP, survivin and borealin, Aurora-B is localized at the centromere and the spindle mid-zone, where it has key roles in the regulation of kinetochore–microtubule attachment, spindle assembly checkpoint (SAC) activation and cytokinesis [5, 6].

In recent years, Aurora-B has been demonstrated to be involved in the recruitment of SAC components during early mitosis. A positive feedback loop between Aurora-B and Mps1 is required for rapid assembly of centromere and kinetochore proteins [5,7–9]. Importantly, the centromere accumulation of CPC is required for the efficiency of the Aurora-B–Mps1 feedback loop [6, 7, 9]. In early mitosis, the centromeric localization of CPC is initially mediated by survivin, which recognizes the phosphorylation of histone H3 threonine 3 (H3T3-ph) and anchors the CPC to the chromatin including centromere [10–13]. Currently, Haspin is the only kinase found to perform H3T3-ph [13, 14]. In late G2 phase and prophase, cyclin-dependent kinase 1 (CDK1) and Plk1 phosphorylate Haspin at multiple sites, which triggers its activation and may release it from autoinhibitory conformation [15, 16]. In prophase, phosphorylation of H2A threonine 120 (H2AT120ph) and the signaling axis: Bub1-H2AT120ph-Sgo, also promote the centromere establishment of CPC at kinetochore proximal side [5, 11, 12].

Although Aurora-A has been shown to be involved in the regulation of the SAC in mitosis, contradictory results have been reported. It was shown that Aurora-A is not required for checkpoint signaling because the key checkpoint protein Mad2 shows robust signals at kinetochores after Aurora-A depletion in mouse embryonic fibroblast (MEF) cells [17]. In addition, SAC was proficient in Aurora-A knockout (KO) DT40 cells [18]. However, Aurora-A was shown to regulate the SAC inactivation by phosphorylating p73, a member of p53 family [19]. Furthermore, when cancer cell HCT116 was treated with the Aurora-A inhibitor MLN8054 and spindle poisons, the cells were only transiently arrested in mitosis, indicating that SAC was compromised [20]. Another study also showed that Aurora-A was required for the maintenance of SAC when cells were treated with microtubule poisons [21]. Moreover, recent work demonstrated Aurora-A is involved in destabilizing erroneous kinetochore–microtubule attachment around the spindle poles [22, 23].

Despite that Aurora-A has been extensively studied, whether Aurora-A has a role in the regulation of the SAC remains elusive. Additionally, although Aurora-A begins to enter nucleus several hours before mitosis [4, 24, 25], the functions of nuclear Aurora-A in early mitosis remain unclear. In the present study, we revealed a role of nuclear-accumulated Aurora-A in the initial establishment of the CPC and the SAC from late G2 to prophase. Using different approaches, including selective inhibitors, genetic KO and ectopic expression, we demonstrate that Aurora-A phosphorylates and activates Haspin kinase independently of Plk1 to facilitate histone H3T3-ph, which promotes timely assembly of CPC and SAC proteins before nuclear envelope breakdown (NEBD). These data reveal that, during the G2–M transition, nuclear-accumulated Aurora-A triggers the feedback loop of Haspin-H3T3-ph-CPC, which ensures the timely establishment of mitotic surveillance mechanisms.

## Results

### Aurora-A is required for the initial recruitment of SAC proteins

**Figure 1:**
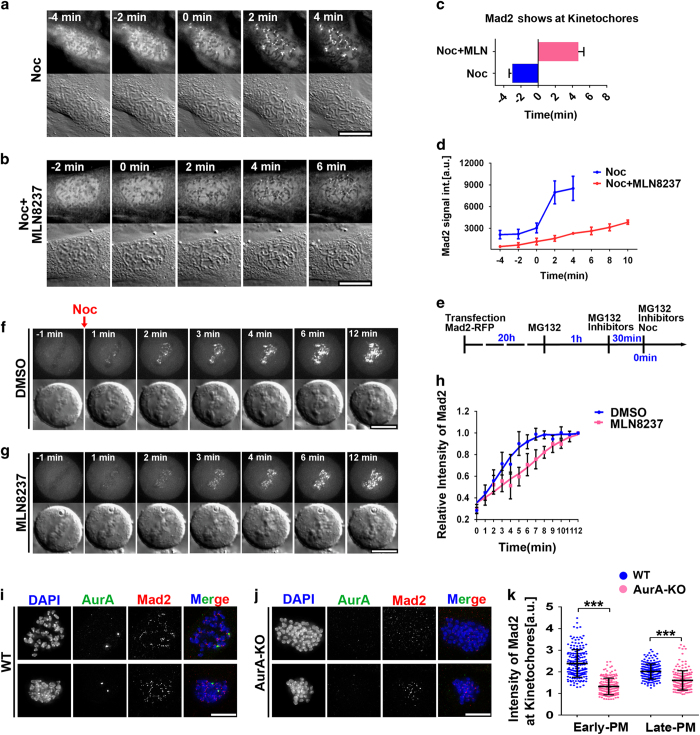
Aurora-A kinase activity is required for the rapid recruitment of checkpoint protein to kinetochores in early mitosis or during checkpoint recovery. (**a**, **b**) Ptk2 cells stably expressing YFP-Mad2 were treated with nocodazole (Noc, 10 μM) (**a**) or Noc+MLN8237 (MLN, 100nM) (**b**). Live cells in prophase were filmed using both different interference contrast (DIC) and fluorescence microscopy to monitor the dynamics of Mad2 recruitment to the kinetochore. Time 0 is defined as the last time point before massive movements of condensed chromosomes were observed. (**c**) Statistics of the first time point when Mad2 signal appears at kinetochore in **a**, **b** (*n*=12). (**d**) Quantification of the intensity of Mad2 at the kinetochore in cells treated with Noc or Noc+MLN is plotted over time (*n*=12). (**e**) The procedure of the checkpoint recovery assays (detailed in Materials and Methods section). (**f**, **g**) Hela cells expressing Mad2-RFP were treated as shown in **e**. Mad2 fluorescence and DIC were monitored in live cells exposed to dimethyl sulfoxide (DMSO) (**f**) and MLN8237 (50 nM) (**g**). (**h**) The quantification of Mad2 intensities at kinetochores is illustrated for cells treated with DMSO (*n*=10) or MLN8237 (*n*=10). Four independent experiments were performed. (**i**, **j**) Mad2 staining in Aurora-A wild-type (WT) and Aurora-A knockout (KO) cells are shown. Cell in early prometaphase is shown in top panel and cells in late prometaphase is shown in bottom panel. (**k**) Quantification of Mad2 intensity in early prometaphase (PM) or late prometaphase cells. Mouse embryonic fibroblast cells (*n*=30–40) from four independent embryos were examined. Data are presented as mean±s.d., ***P<0.001 (Student’s *t*-test). a.u., arbitrary unit; DAPI, 4,6-diamidino-2-phenylindole. Scale bar: 10 μm.

Aurora-A is accumulated in the nucleus from late G2 to prophase. Meanwhile, the SAC proteins must be rapidly recruited to kinetochores in the nucleus before NEBD [7, 9]. Therefore, we wondered whether nuclear Aurora-A contributes to the establishment of the SAC in late G2 and prophase. We first visualized the initial recruitment of Mad2, one of the key components of the SAC, to the kinetochores in live cells with or without Aurora-A kinase activity. Mad2 signals were monitored from prophase to early prometaphase in Ptk2 cells that stably expressed Mad2-YFP (Figure 1a). Microtubules were completely depolymerized with nocodazole (Noc) to prevent Mad2 being dissociated from the kinetochore upon attached to the microtubules after NEBD. In control cells, Mad2 was first detected at kinetochores approximately 3 min before NEBD (*T*=0 at NEBD, defined as the time point before massive movements of condensed chromosomes were observed) (Figure 1a and c), whereas in MLN8237 (an inhibitor that is highly selective for Aurora-A [26]) treated cells Mad2 appeared at kinetochores approximately 5 min after the NEBD (Figure 1b and c). Additionally, the intensity of the Mad2 signal at kinetochores was much lower in the Aurora-A-inhibited cells (Figure 1d). Furthermore, the rate of Mad2 recruitment was also markedly decreased when Aurora-A kinase activity was inhibited (Figure 1d).

To confirm whether Aurora-A is involved in the initial recruitment of checkpoint proteins, a checkpoint recovery assay was conducted [27]. Briefly, HeLa cells expressing Mad2-RFP were arrested in metaphase using MG132. When Mad2-RFP fluorescence disappeared from all the kinetochores, the Aurora-A inhibitor or dimethyl sulfoxide (DMSO) was added, followed by adding Noc to reactivate the recruitment of Mad2 to the kinetochores [27] (Figure 1e). In the DMSO-treated cells, Mad2 was fully recovered at the kinetochore within 5 min (Figure 1f and h), while recovery was much slower (~11 min) in the MLN8237-treated cells (Figure 1g and h). Nonetheless, after recovery the level of Mad2 at the kinetochore was not prominently affected by Aurora-A inhibition (Figure 1g), which is in agreement with the result that Aurora-A kinase inhibition does not prevent SAC activation [17]. Notably, this delay was not due to reduction of Aurora-B kinase activity, as in MLN8237-treated cells, the kinase activity of Aurora-B was just slightly affected (Supplementary Figure S3a). Taken together, these results revealed that Aurora-A kinase activity is crucial for the rapid recruitment of SAC proteins to kinetochores during early mitosis.

To verify the function of Aurora-A in the recruitment of SAC proteins, we examined the localization of Mad2 at different stages of mitosis in Aurora-A KO MEF cells that were generated from conditional-KO embryos [17] (Materials and Methods section). In late prometaphase cells (whose chromosomes gathered at the center of the cell), the intensity of Mad2 at kinetochores was decreased by ~15% upon Aurora-A depletion as previously described (bottom row, Figure 1j and k) [17]. However, in early prometaphase cells (whose chromosomes scattered [7] without extensive movements, top row in Figures 1i and j), the level of Mad2 was considerably reduced by ~52% in the Aurora-A KO MEFs comparing to that in the wild-type (WT) MEFs (Figure 1i–k). The level of other SAC proteins, including BubR1, Mad2 and Bub1, at kinetochores were also examined in nontransformed hTERT-RPE1 (RPE1) cells that were treated with MLN8327. Consistently, the levels of Mad2, BubR1 and Bub1 at kinetochore were significantly reduced when Aurora-A activity was inhibited in prophase or early prometaphase cells, whereas the reductions were less significant in late prometaphase cells (Supplementary Figure S1a–h). The reduction of Bub1 at kinetochore is less significant in Aurora-A-inhibited than in Mps1-inhibited cells with Reversine (Supplementary Figure S1g and h).

In late G2 phase, Mad2 is recruited to the nuclear pore complex where it is activated as an inhibitor of Cdc20 [28], and this process is crucial for a fully functional SAC in mitosis [28]. Therefore, we investigated whether Aurora-A is also required for the distribution of Mad2 at the nuclear pore in G2 phase. The results showed that this part of Mad2 was not affected when Aurora-A was inhibited (Supplementary Figure S2). Thus these data demonstrate that Aurora-A inhibition delays the early recruitment of SAC proteins to the kinetochores from late G2 to early prometaphase.

### Aurora-A regulates the SAC establishment by modulating centromere recruitment of CPC in early mitosis

**Figure 2:**
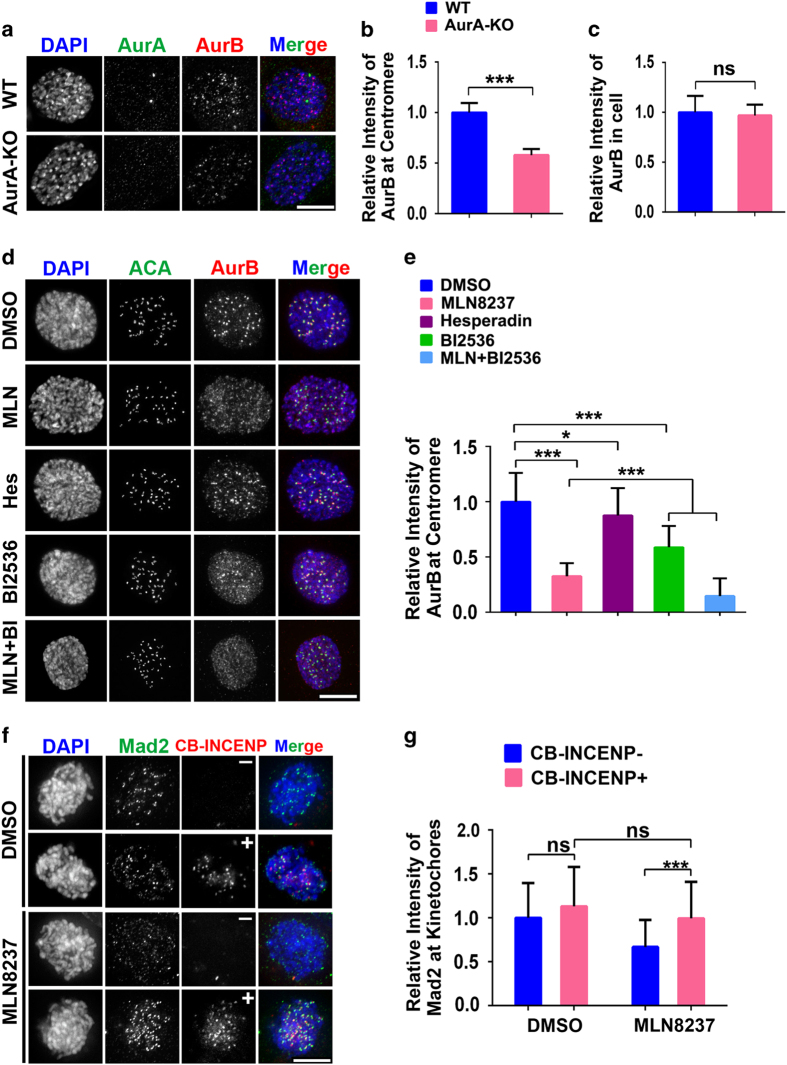
Aurora-A regulates the recruitment of the spindle assembly checkpoint protein through promoting Aurora-B to centromeres. (**a**) Aurora-B was stained in Aurora-A wild-type (WT) or knock-out (KO) mouse embryonic fibroblast (MEF) cells in prophase. MEF cells (*n*=30–40) were examined in three independent experiments. (**b**, **c**) Quantification of the intensity of Aurora-B at centromere (**b**) or in the whole cell (**c**) in MEF cells in prophase. (**d**) RPE1 cells were treated with inhibitors for Aurora-A: MLN8237 (MLN), Aurora-B: hesperadin (hes) and Plk1: BI2536 for 2 h. Aurora-B and ACA were co-labeled in prophase cells. (**e**) The intensity of Aurora-B fluorescence at the centromere is quantified. Cells (*n*=90–100) were analyzed from three independent experiments. (**f**) Hela cells transfected with CENPB-INCENP-mCherry (CB-INCENP) were treated with dimethyl sulfoxide (DMSO) or MLN8237 for 2 h. Mad2 at kinetochore was monitored. (**g**) Quantification of Mad2 intensity in **f**; 200 kinetochores from 40 cells were included. Four independent experiments were performed. Data are represented as mean±s.e.m. *P<0.05; ***P<0.001 (Student’s *t*-test), DAPI, 4,6-diamidino-2-phenylindol; NS, not significant. Scale bar: 10 μm.

Because Aurora-B/CPC is responsible for the rapid assembly of the SAC proteins [7, 9], we sought to explore whether Aurora-A is involved in the establishment of the SAC by regulating Aurora-B/CPC during the G2–M transition. Indeed, the level of Aurora-B at centromere was significantly reduced in Aurora-A KO MEF cells in prophase (Figure 2a and b). However, the total level of Aurora-B was not disturbed (Figure 2c). Consistently, in RPE1 cells treated with MLN8237, the accumulation of Aurora-B from chromosomal arms to centromeres was significantly diminished (by ~70%) in prophase cells (Figure 2d and e). Because the kinase activities of Aurora-B and Plk1 are required for the full recruitment of CPC at centromere [8,  16, 29–31], and Aurora-A phosphorylates and activates Plk1 in early mitosis [25, 32, 33], it is possible that Aurora-A modulates the recruitment of CPC by activating Aurora-B or Plk1. However, the reduction of Aurora-B at centromeres was less (<50%) in the Aurora-B (Hesperadin-treated) or Plk1 (BI2536-treated) inhibited cells than that in Aurora-A-inhibited cells (Figure 2d and e). In addition, the synergistic effect of combination of Aurora-A and Plk1 inhibition on Aurora-B recruitment (Figure 2d) suggest that Aurora-A regulates the recruitment of Aurora-B in Plk1-independent manners. Notably, phosphor-Plk1 status was examined with Plk1 T210ph antibody. The result from immune-blotting indicated that Plk1 activity was slightly reduced by MLN8237 (Supplementary Figure S3a). Consistently, immune-staining results with Plk1 T210ph antibody indicated that Plk1 activity was only moderately affected at kinetochore/centrosome in prophase (Supplementary Figure S3b–f). Thus these data suggest that in late G2 phase Aurora-A is required for the recruitment of Aurora-B and this function of Aurora-A is largely independent on kinase activity of Aurora-B or Plk1.

Next we explored whether Aurora-A regulates the assembly of the SAC proteins primarily by modulating the recruitment of CPC to centromeres. We performed a rescue assay, in which CPC was constitutively anchored to centromeres by the fusion protein: CENPB-INCENP-mCherry (CB-INCENP; CENPB binds to satellite DNA at centromere) [34]. In untransfected cells, inhibiting Aurora-A delayed the recruitment of Aurora-B to centromeres in early mitosis. Whereas in cells expressing CB-INCENP, the centromere localization of Aurora-B was largely undisturbed upon Aurora-A inhibition (Supplementary Figure S4a and b). We then examined whether the recruitment of checkpoint proteins could be rescued by CB-INCENP when Aurora-A was inhibited. Indeed, in CB-INCENP-transfected cells, Mad2 was unequivocally localized to kinetochores in early prometaphase cells in the presence of MLN8237 (Figure 2f and g). These results suggest that Aurora-A regulates SAC protein assembly primarily by facilitating the initial recruitment of the CPC to centromeres during early mitosis.

### Aurora-A modulates H3T3-ph during early mitosis

**Figure 3:**
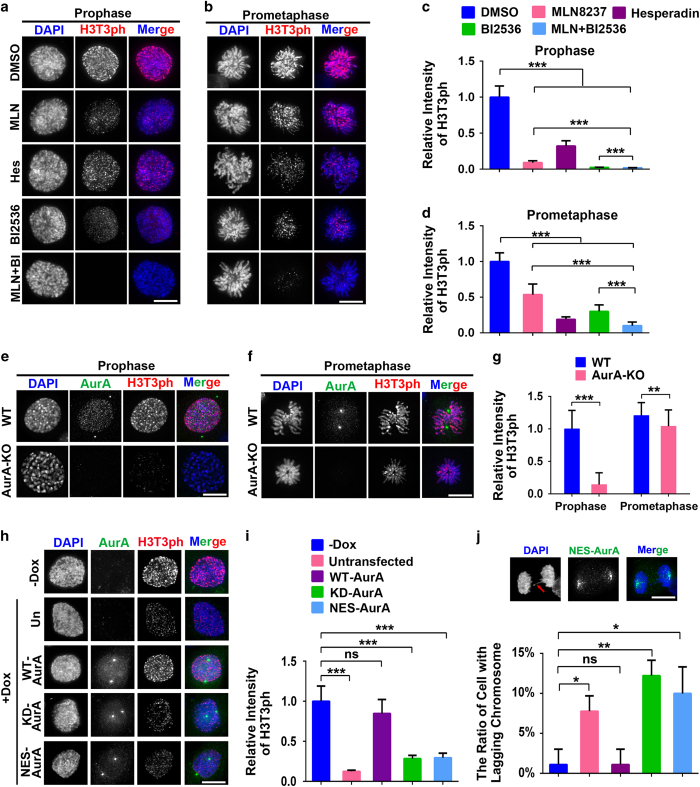
Nuclear Aurora-A is crucial for H3T3-ph in early mitosis. (**a**, **b**) RPE1 cells were treated with inhibitors for Aurora-A (MLN, 50 nM), Aurora-B (Hes, 100 nM) and Plk1 (BI2536, 100 nM) for 2 h. The H3T3-ph was examined in cells in prophase (**a**) and prometaphase (**b**). (**c**, **d**) Quantification of the intensity of H3T3-ph signal in prophase (**c**) and prometaphase (**d**) cells, *n*=100. (**e**, **f**) The H3T3-ph was stained in the Aurora-A wild-type (WT) and knockout (KO) mouse embryonic fibroblast cells in prophase (*n*=97) (**e**) and prometaphase (*n*=107) (**f**). (**g**) Quantification of the H3T3-ph intensity in **e**, **f**. (**h**) Endogenous Aurora-A was knocked down by doxycycline-induced shRNA (+dox), while shRNA-resistant green fluorescent protein-tagged WT, kinase deficient (KD) Aurora-A and nuclear export signal (NES)-Aurora-A were transfected in Hela cells. H3T3-ph is examined by immunostaining. (**i**) Quantification of the H3T3-ph intensity in **h**. *n*=60. (**j**) Typical image (above) and ratio (below) of anaphase lagging chromosomes in NES-Aurora-A-transfected cells. *n*=90. Three or four independent experiments were performed. Data are represented as mean±s.e.m. *P<0.05 (Student’s *t*-test); **P<0.01 (Student’s *t*-test); ***P<0.001 (Student's *t*-test), DAPI, 4,6-diamidino-2-phenylindol; DMSO, dimethyl sulfoxide; NS, not significant. Scale bar: 10 μm.

It has been proved that histone modification H3T3-ph is the early event that recruits CPC/Aurora-B in late G2 phase [16]. We reasoned that Aurora-A may function in CPC recruitment by regulating H3T3-ph levels. Therefore H3T3-ph was examined by immunofluorescence in RPE1 cells. In control prophase cells, H3T3-ph was robustly distributed both on chromosome arms and centromeres [14] (Figure 3a). However, in prophase cells treated with the Aurora-A inhibitor, the total intensity of H3T3-ph was significantly reduced to <20% of the level observed in uninhibited cells (Figure 3a and c), indicating that Aurora-A is involved in the regulation of H3T3-ph. Similar results were obtained in Plk1-inhibited (BI2536-treated) cells (Figure 3a and c), which is in line with the results in the previous report [15, 16], whereas in cells treated with the Aurora-B inhibitor hesperadin, the level of H3T3-ph was reduced to approximately 40% in cells in late G2 phase (Figure 3a and c). When Aurora-A and Plk1 were simultaneously inhibited, the level of H3T3-ph was nearly completely abolished (Figure 3a and c). This synergetic effect indicates that this function of Aurora-A is at least partially independent of Plk1 in promoting H3T3-ph. In prometaphase cells, the reduction of H3T3-ph was weaker than the reduction observed in prophase cells when Aurora-A was inhibited (Figure 3b and d) but greater than the reduction observed when Aurora-B was inhibited. These data suggest that Aurora-A modulates H3T3-ph mainly in early mitosis, whereas Aurora-B has a more important role in promoting H3T3-ph in later stages. Consistently, in Aurora-A KO MEF cells, the level of H3T3-ph was clearly reduced in late G2-prophase (Figure 3e and g), whereas this reduction was much weaker in prometaphase cells (Figure 3f and g). Similar result of reduction of H3T3-ph after Aurora-A inhibition was also obtained in human cancer cell line A549 (Supplementary Figure S5d and e).

To evaluate the contribution of nuclear-localized Aurora-A to the H3T3-ph in late G2 phase, a nuclear export signal (NES)-tagged Aurora-A [35], kinase deficient Aurora-A (KD) and wild type Aurora-A (WT) were expressed (Supplementary Figure S5b) while endogenous Aurora-A was depleted with shRNA (Supplementary Figure S5a). Centrosome separation was not disturbed when the nuclear accumulation of Aurora-A was blocked by NES-Aurora-A in late G2 phase (Figure 3h), suggesting that NES-Aurora-A did not disturb either the Aurora-A kinase function at centrosomes or its general kinase activity. Remarkably, compared with uninduced or WT Aurora-A-transfected cells, in the NES-Aurora-A or KD-Aurora-A (kinase deficient) positive cells the H3T3-ph signal was dramatically decreased in late G2 phase (Figure 3h and i). Therefore, this result confirmed the hypothesis that kinase activity of nuclear Aurora-A is involved in the efficient H3T3-ph in late G2 and prophase. It is worth mentioning that the ratio of lagging chromosome increases from 1% in WT Aurora-A-expressing cells to over 10% in NES-Aurora-A-expressing cells (Figure 3j) and the number of binucleated cells was also increased (Supplementary Figure S5c). These data suggest that nuclear accumulation of Aurora-A in late G2 cells is crucial for the accuracy of chromosome segregation.

### Aurora-A-mediated H3T3-ph is independent of CPC

**Figure 4:**
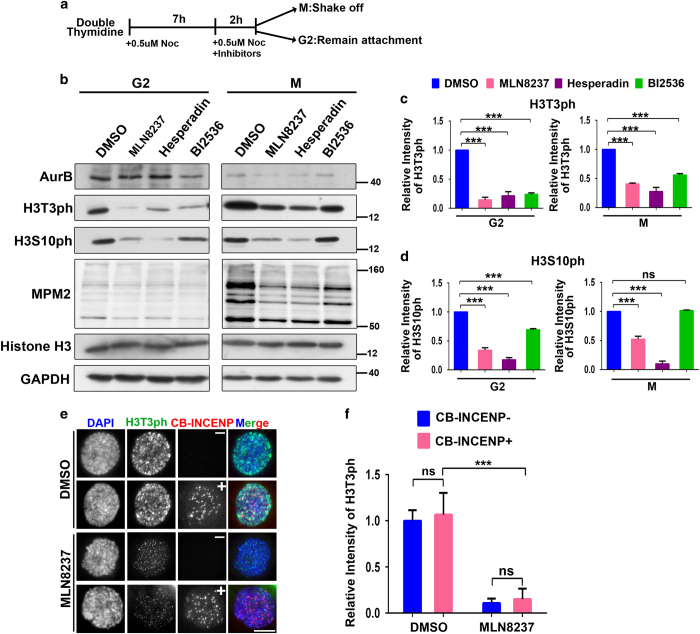
Aurora-A modulates H3T3-ph in a chromosomal passenger complex (CPC)-independent manner. (**a**) Diagram showing the immunoblotting assay performed to examine H3T3-ph levels. Cells were synchronized by releasing from double thymidine block, M phase cells were collected by shake-off 9 h after release and the attached cells after shaking-off were regarded to be cells in G2 phase. (**b**) H3T3-ph and H3S10-ph level were monitored using immunoblotting in synchronized Hela cells treated with Aurora-A (50 nM), Aurora-B (100 nM) and Plk1 (100 nM) inhibitors. (**c**, **d**) Quantification of the level of H3T3-ph (**c**) and H3S10ph (**d**) in **b**. Three independent experiments were performed. (**e**) Tethering CPC at centromeres failed to rescue H3T3-ph upon Aurora-A inhibition. Hela cells transfected with CB-INCENP were treated with dimethyl sulfoxide (DMSO) or MLN8237 for 2 h. H3T3-ph was monitored with immunostaining. (**f**) H3T3-ph in **e** is quantified. *n*=60, three independent experiments were performed, Values represent as mean±s.e.m. ***P<0.001 (Student’s *t*-test), DAPI, 4,6-diamidino-2-phenylindol; GAPDH, glyceraldehyde 3-phosphate dehydrogenase; NS, not significant. Scale bar: 10 μm.

The level of H3T3-ph was also evaluated using western blotting analysis in synchronized HeLa cells upon Aurora-A inhibition (procedure shown in Figure 4a). In G2-phase cells, the level of MPM2 epitope (the mitotic phospho-epitope) was low and was not affected by the Aurora or Plk1 inhibitors (Figure 4b), suggesting that the cells were well synchronized and that the mitotic population was not changed upon kinases’ inhibitions. Remarkably, in the lysate of G2 cells, the total H3T3-ph was dramatically reduced by the Aurora-A inhibitor (Figure 4b and c). This inhibitory effect was partially restored in prometaphase cells (Figure 4b and c), which is consistent with the results obtained in immunostaining experiments (Figure 3a–d). Thus these results support the hypothesis that Aurora-A kinase is required for H3T3-ph mainly in late G2 phase and that it contributes much less in prometaphase.

As both Aurora-A and Aurora-B promote H3T3-ph (Figures 3 and 4a–c), [8], and Aurora-A kinase activity is required for the centromere recruitment of Aurora-B (Figure 2a–d), it is possible that Aurora-A modulates H3T3-ph via recruiting Aurora-B/CPC. If this is the case, constitutively active CPC should rescue the H3T3-ph signal upon Aurora-A inhibition. However, in CB-INCENP-expressing cells, the H3T3-ph was as low as it was in untransfected cells when Aurora-A was inhibited with MLN8237 (Figure 4e and f), whereas the H2AT120-ph was mostly restored in CB-INCENP-expressing cells treated with MLN8237 (Supplementary Figure S4c and d), suggesting that Aurora-A regulates H2AT120-ph through recruiting CPC, while Aurora-A regulates H3T3-ph via a CPC-independent pathway in late G2 phase.

### Aurora-A phosphorylates Haspin and regulates its kinase activity

**Figure 5:**
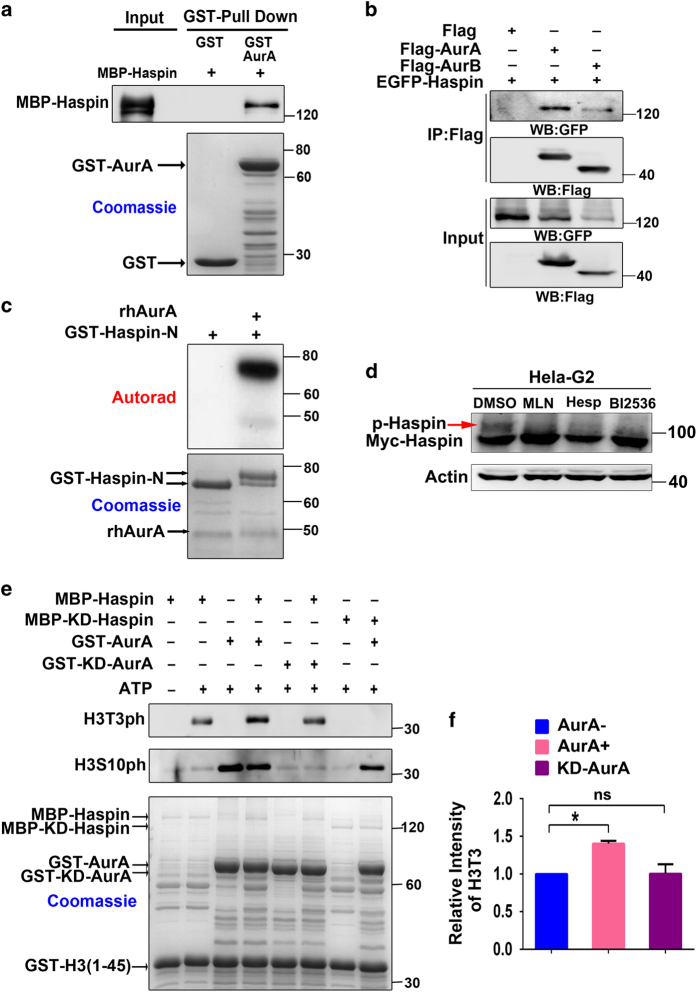
Aurora-A directly interacts with and phosphates Haspin in early mitosis. (**a**) Glutathione *S*-transferase (GST)-fused Aurora-A and maltose-binding protein (MBP)-fused Haspin protein were expressed in *Escherichia coli* and then purified. GST pull-down assays were performed. MBP-Haspin was detected with a Haspin antibody. (**b**) Flag-tagged Aurora-A, Aurora-B and the green fluorescent protein (GFP)-fused Haspin were co-expressed in 293T cells. Immunoprecipitation (IP) was performed using Flag antibody-conjugated beads. The GFP-Haspin protein was detected using a GFP antibody. (**c**) *In vitro* kinase assays were performed to demonstrate that rhAurora-A directly phosphorylates Haspin-N. (**d**) Uninduced HeLa Tet-On/myc-Haspin cells[8] were treated with MLN8237 (MLN, 50 nM), Hesperadin (Hesp, 100 nM) and BI2536 (100 nM). Cells in G2 phase were collected with the protocol in Figure 4a. The shifted band for phosphorylated Haspin were detected by western blotting (WB) with Myc (4A6) antibody. (**e**) Purified MBP-Haspin, GST-Aurora-A and GST-H3 (1-45) were mixed as indicated. Mixture was incubated with ATP for 30 min to allow proteins to be phosphorylated and then the level of H3T3-ph and H3S10-ph was detected by WB. (**f**) Quantification of H3T3-ph level in **e**. Three independent experiments were performed. Values represent mean±s.e.m. *P<0.05 (Student’s *t*-test), NS, not significant.

As Aurora-A regulates H3T3-ph through a CPC-independent pathway, it is tempting to assume that Aurora-A directly binds and phosphorylates Haspin to promote H3T3-ph in the nucleus in late G2 phase. As expected, the result from the pull-down assay performed with glutathione *S*-transferase (GST)-Aurora-A and maltose-binding protein (MBP)-Haspin revealed that Aurora-A directly interacted with Haspin *in vitro* (Figure 5a). Data from co-immunoprecipitation with green fluorescent protein (GFP)-Haspin and FLAG-Aurora-A in HEK293T cells revealed that Aurora-A was associated with Haspin *in vivo* (Figure 5b). Next we sought to explore whether Aurora-A also phosphorylates Haspin directly. A kinase assay was performed using recombinant Aurora-A and GST-fusion Haspin-N (1–350 aa), which includes most of the Aurora-B phosphorylation sites (also consensus phosphorylation sites for Aurora-A) [8]. Haspin-N lacks the kinase domain and therefore does not display self-phosphorylating activity. Further, Haspin-N displays same nucleus localization as full-length Haspin does in late G2 phase (Supplementary Figure S6a). Autoradiography results showed that Haspin-N was strongly phosphorylated by Aurora-A (Figure 5c). Notably, GST-Haspin-N exhibited super-shift bands after it was incubated with recombinant human Aurora-A (rhAurora-A) (Figure 5c), suggesting that Haspin-N was highly phosphorylated by Aurora-A. Additionally, the phosphorylated Haspin-N was separated and analyzed using liquid chromatography–mass spectrometry to identify phosphorylation sites. Five Serine sites (S93, S108, S143, S147 and S216) were detected (Supplementary Figure S6b), and these sites were shown to correspond to Aurora-B phosphorylation sites previously identified in mitotic cells [8]. Co-localization was observed between Aurora-A and GFP-Haspin in the nucleus in G2 phase (Supplementary Figure S6c). Furthermore, the reduced migration-shift band of phosphorylated Haspin in G2 phase after Aurora-A inhibition reveals that Aurora-A phosphorylates Haspin *in vivo* (Figure 5d). Thus these results indicate that Aurora-A directly phosphorylates Haspin at multiple sites that are also phosphorylated by Aurora-B.

To investigate whether Aurora-A-mediated phosphorylation is associated with Haspin activity, phosphor-mimic mutant EGFP-Haspin 11E [8] and WT Haspin were used to rescue H3T3-ph level in the presence of Aurora-A inhibitor. EGFP-Haspin 11E showed evidently higher activity in phosphorylating H3T3 than WT Haspin did after Aurora-A was inhibited (Supplementary Figure S6d and e), which implied that phosphorylation at these sites promotes Haspin activity. Moreover, the ability of MBP-Haspin in phosphorylating GST-H3 (1-45) at Thr3 was considerably enhanced after it was preincubated with WT Aurora-A but not KD-Aurora-A (Figure 5e and f), suggesting that Aurora-A activates Haspin by direct phosphorylation. Altogether, these data suggest that Aurora-A promotes Haspin kinase activity by direct phosphorylation.

### Aurora-A promotes the interaction between Aurora-B and Haspin in early mitosis

**Figure 6:**
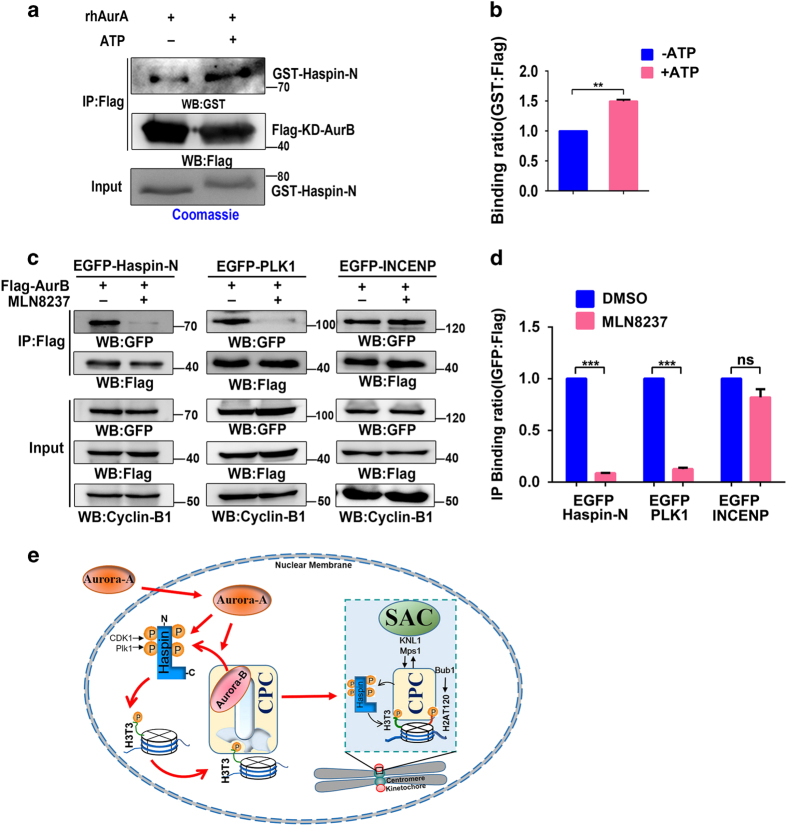
Aurora-A promotes the association of Aurora-B with Haspin and Plk1. (**a**) Flag-tagged kinase deficient (KD)-Aurora-B was expressed and immunoprecipitated (IP) in 293T cells. glutathione *S*-transferase (GST)-fused Haspin-N proteins were preincubated with human recombinant Aurora-A (rhAurA) with or without ATP and were mixed with KD-Aurora-B on FLAG-Beads. Pull-down assay was conducted with FLAG beads, and Haspin-N was detected with GST antibody. (**b**) A statistical analysis of three experiments is shown for **a**. (**c**) Flag-tagged Aurora-B was co-expressed with green fluorescent protein (GFP)-Haspin-N, Plk1 or INCENP. Two hours before harvest, cells were treated with 0.5 μM nocodazole, and Aurora-A inhibitor or dimethyl sulfoxide (DMSO) was added as indicated. Co-IP was performed with FLAG beads. (**d**) The IP-binding ratio (GFP: FLAG) for precipitates in **c** is quantified. Three or four independent experiments were performed. Values represent mean±s.e.m. **P<0.01; ***P<0.001; NS: not significant (Student’s *t*-test). (**e**) A model is shown to illustrate the function of Aurora-A in promoting Haspin-H3T3-ph-CPC (chromosomal passenger complex) feedback loop in early mitosis. See the Discussion section for a detailed description. EGFP, enhanced GFP; SAC, spindle assembly checkpoint; WB, western blotting.

As Aurora-A and Aurora-B phosphorylate Haspin at the same sites, we wondered whether these two kinases regulate each other in association with Haspin. Interestingly, the interaction between Aurora-B and Haspin was enhanced if Haspin was phosphorylated by rhAurora-A *in vitro* before mixing with Aurora-B (Figure 6a and b). Moreover, results from a co-immunoprecipitation assay indicated that the association of Aurora-B with Haspin and Plk1 were significantly attenuated when Aurora-A was inhibited, whereas the interaction between Aurora-B and CPC component INCENP was not affected (Figure 6c and d), suggesting that Aurora-A promotes the association of Aurora-B with Haspin and Plk1. Taken together, these data demonstrate that Aurora-A promotes H3T3-ph by directly phosphorylating and activating Haspin, which is essential for the efficient recruitment of CPC to centromeres. Additionally, Aurora-A promotes the association of Aurora-B with Haspin and Plk1, which may further facilitate the positive feedback loop of Haspin-H3T3-ph-CPC in early mitosis.

## Discussion

As there is no quality control that monitors the integrity of the SAC assembly, cells must evolve highly efficient ways to ensure the rapid generation of the SAC before NEBD, after which the chromosomes are immediately encountered with highly dynamic microtubules. A delay in the establishment of these surveillance machineries causes erroneous microtubule–kinetochore attachments and subsequent induction of chromosome mis-segregation, which could eventually lead to malignant transformation [7, 36]. It is known that CPC at centromere is responsible for the efficient establishments of both the error-correction machinery and the SAC [5–7]. However, how initial recruitment of CPC to the centromere is regulated remains elusive.

It is established that Aurora-A promotes G2–M transition by activating Cdk1/cyclin-B and Plk1 at centrosome [37]. In addition to its known functions at centrosomes, activated Aurora-A kinase is accumulated in the nucleus from late G2 phase to prophase. It is therefore reasonable to assume that Aurora-A is involved in triggering events that is responsible for the establishment of surveillance mechanisms in the nucleus before NEBD. In the current study, we demonstrate that during early mitosis Aurora-A directly phosphorylates Haspin kinase at multiple sites to promote Haspin activity and H3T3-ph, which facilitates the recruitment of CPC to centromeres. Furthermore, we show that Aurora-A promotes the association of Aurora-B with Haspin and Plk1. These functions of Aurora-A prime the positive feedback loop of Haspin–H3T3-ph–Aurora-B [8] and ensure the rapid assembly of CPC at centromeres and checkpoint proteins at kinetochores during early mitosis. This signaling pathway during early mitosis is crucial for the accurate chromosome segregation (Figure 3j and Supplementary Figure S5c). The mechanisms underlying the activation of Haspin and the increased association of Haspin with Aurora-B upon Aurora-A phosphorylation need to be further explored. Possible explanation is that phosphorylation of Haspin by Aurora-A prevents Haspin from autoinhibition [15] or from association with Phosphatase 1 that inactivates Haspin [38, 39].

Plk1 has been shown to promote Haspin kinase and H3T3-ph in prophase, which facilitates the recruitment of CPC [15, 16]. Furthermore, Aurora-B activity at centromere was proved to be modulated by Plk1-dependent survivin phosphorylation [29]. Recently, Plk1 was also reported to regulate the SAC establishment in combination with Mps1 in *Caenorhabditis elegans*, *Drosophila* and human cells [40–42]. Because Plk1 is activated by Aurora-A accompanying with Bora during the G2/M transition and mitosis [25, 32, 33], it is possible that Aurora-A modulates H3T3-ph as well as the SAC establishment mainly by activating Plk1. However, several lines of evidence indicate that this is not the case. First, Aurora-A directly binds to and phosphorylates Haspin, suggesting that it has a direct role in modulating Haspin kinase (Figure 5). Second, when the delay in the establishment of the SAC upon Aurora-A and Plk1 inhibition were compared, the reduction in Mad2 and BubR1 during early prometaphase was weaker in Plk1-inhibited than in Aurora-A-inhibited cells (Supplementary Figure S1b–d). These data indicate that the function of Aurora-A in CPC recruitment in early mitosis is at least partially independent of Plk1. Third, the Plk1 inhibitor BI2536 and the Aurora-A inhibitor MLN8237 had a synergetic effect in reducing Aurora-B and H3T3-ph levels (Figures 2d, e and 3a–d), which implies that the function of Aurora-A in CPC recruitment is not performed merely by activating Plk1. Moreover, in checkpoint recovery assays (Figure 1f), although Plk1 activity was no longer significantly disturbed when Aurora-A was inhibited in late prometaphase cells [25], the re-establishment of Mad2 at kinetochores was still slowed down. This result also suggests that the functions of Aurora-A in initial recruitment of checkpoint proteins involve processes other than activating Plk1. Nevertheless, we do not exclude the possibility that delay of Plk1 activation [25, 32, 33] and moderate decrease of Plk1 kinase activity after Aurora-A inhibition (Supplementary Figure S3) contribute to the phenotypes in this study. Notably, it was reported that Plk1 inhibition also disturbs Aurora-A function by regulating the degradation of hBora [43]. In fact, we also noticed that BI2536 treatment inhibited Aurora-A kinase activity in mitosis (Supplementary Figure S3a), which reveals mutual regulation of Aurora-A and Plk1 in mitosis. We therefore suggest that both Aurora-A and Plk1 are involved in the activation of Haspin kinase by phosphorylating different sites.

The Aurora-A and -B kinases have been shown to phosphorylate common substrates at the same sites [2, 4, 22, 23, 38]. However, whether the sites of Haspin phosphorylated by Aurora-A and Aurora-B are the same need to be further confirmed. Importantly, in this study we demonstrated that, by priming Haspin phosphorylation, Aurora-A promotes not only the recruitment of Aurora-B but also the association of Aurora-B with Haspin and Plk1 during early mitosis. After centromere recruitment of CPC is established during prometaphase, those substrates are mainly phosphorylated by Aurora-B via a physical interaction at centromeres. Aurora-A kinase activity is then no longer required for CPC localization or checkpoint protein recruitment. These results implicate that Aurora kinases work together in one feedback loop during early mitosis when both of them reside in the nucleus. We propose that this is an efficient way in which two Aurora kinases work simultaneously and synergistically during early mitosis.

Because Aurora-B kinase activity is boosted by the centromeric localization of CPC itself [5, 44, 45], Aurora-A inhibition could indirectly reduce Aurora-B activity by lessening the recruitment of CPC (Figure 2), which may contribute to the compromised activity of the feedback loop of Haspin–H3T3-ph–Aurora-B (Figures 2,2,3,4,5,6). This may also partially explain the reduction of H3S10ph upon Aurora-A inhibition (Figure 4b), despite that Aurora-A also phosphorylates H3S10 directly [46].

Taken together, we propose a model (Figure 6e) for the function of nuclear Aurora-A in late G2 phase: active Aurora-A accumulates in the nucleus and phosphorylates Haspin to prime H3T3-ph and a positive feedback of Haspin–H3T3-ph–Aurora-B. This is a new pathway that is parallel to Cdk1–Plk1 pathway in Haspin initial activation [15, 16]. Moreover, nuclear Aurora-A facilitates the association of Aurora-B with Haspin and Plk1 (Figure 6c), which also expedites the formation of the Haspin–H3T3-ph–Aurora-B feedback loop that is essential for the efficient recruitment of Aurora-B/CPC to centromere (Figure 6e). In prophase, the positive feedback loop between Aurora-B and Mps1 promotes Bub1–H2AT120–Sgo1 axis, which is also crucial for the centromere recruitment of CPC. The fully established CPC ensures the rapid establishment of SAC proteins before NEBD (Figure 6e). Therefore, as one of earliest activated mitotic kinases, nuclear Aurora-A expedites the activation of Haspin and Aurora-B kinases efficiently and promotes the positive feedback loops among mitotic kinases, including Cdk1, Aurora-B, Haspin, Plk1, Mps1 and Bub1 [6, 37]. These positive feedback loops are the basis of the successful establishments of the CPC and SAC. Our work demonstrates that Haspin kinase activation mediated by Aurora-A contributes to these kinase-activation networks.

The Aurora-A kinase inhibitor Alisertib is currently being tested in phases II and III clinical trials [47]. A comprehensive phase II clinical trial recently showed promising results in treatments for advanced lung and breast cancer [48]. The findings of our study suggest that Aurora-A regulates the CPC and SAC during early mitosis, which implies that inhibiting Aurora-A may compromise mitotic surveillance mechanisms and induce aneuploidy in the cycling cells. Thus this function of Aurora-A should be considered for interpreting the efficacy behind the Aurora-A inhibitors in cancer therapies.

## Materials and Methods

### Cell culture

Aurora-A conditional knockout mice and MEF cells were generated as previously described [17]. Aurora-A depletion was induced by 4-OHT according to the protocol described previously [17]. MEF, RPE1, HeLa, HEK293T and Ptk2 cells were cultured in DMEM supplemented with 10% fetal bovine serum (Gibco, Grand Island, NY, USA) and penicillin/streptomycin (100 IU ml^−1^ and 100 mg ml^−1^, respectively; Beyotime Biotechnology, Jiangsu, China). Cells were maintained at 37 °C in a 5% CO_2_ atmosphere and seeded onto coverslips 24–48 h before experimentation. Transfections were performed with Lipofectamine 3000 (Invitrogen, Carlsbad, CA, USA). MLN8237 (50 nM for human cell lines and 100 nM for Ptk2 cells), Hesperadin (100 nM), BI2536 (100 nM), Reversine (500 nM) and Noc (0.5 μM for human cell lines and 3.3 μM for MEF and 10 μM for Ptk2 cells) were purchased from Sigma (St Louis, MO, USA) or Selleck Chemicals (LLC, Houston, TX, USA), and resolved in DMSO.

### Antibodies

Primary antibodies used were rabbit anti-Mad2 (a gift from ED Salmon, Chapel Hill, NC, USA), Rabbit anti-Haspin (a gift from Dr Fangwei Wang, Zhejiang University, China), Mouse anti-BubR1 (BD Transduction, Waltham, MA, USA), Mouse anti-Bub1 (Millipore, Bedford, MA, USA), human anti-centromere (ACA) (Antibodies, Inc., Davis, CA, USA), mouse anti-β-tubulin (Sigma), mouse anti-Aurora A (BD Transduction), rabbit anti-mouse Aurora A (Bethyl, Montgomery, TX, USA), Mouse anti-Aurora B (AIM, BD Transduction), rabbit anti-H3S10ph (Millipore, Bedford, MA, USA), rabbit anti-H3T3ph (Millipore), rabbit anti-H2AT120ph (Active Motif, Carlsbad, CA, USA), mitotic phospho-epitopes (MPM-2; Millipore), rabbit anti-Plk1-pT210 (Epitomics, Cambridge, MA, USA), rabbit anti-AurA-pT288 (Cell Signaling Technology, Danvers, MA, USA), rabbit anti-AurB-pT232 antibody (Abcam, Cambridge, MA, USA), rabbit anti-GFP antibody (Abcam, ab290), rabbit anti-H3 (Abcam, ab1791), mouse anti-GAPDH (TransGen Biotech, Beijing, China), mouse anti-Flag (TransGen Biotech), mouse anti-GST (Proteintech, Wuhan, China). Secondary antibodies used were goat anti-rabbit or mouse HRP, donkey anti-rabbit or mouse Cy5, goat anti-rabbit or mouse Alexa 594, goat anti-human FITC or goat anti-rabbit or mouse Alexa 488 (Jackson Immuno-Research, Westgrove, PA, USA).

### Immunofluorescence

Immunofluorescence was conducted as previously described[49]. For Mad2 and BubR1 staining, cells plated on coverslips were pre-extracted with 0.2% Triton X-100 in PHEM (60 mM PIPES (pH 6.8), 25 mM HEPES, 10 mM EGTA and 2 mM MgCl_2_) for 45 s before fixation with 4% paraformaldehyde in PBS. After staining experiments for Aurora A, Aurora B, H3T3ph, H3S10ph, Bub1 and H2AT120ph, cells were fixed directly in 4% paraformaldehyde before extraction. Then, cells were blocked with 1% bovine serum albumin in TBST for 30 min, incubated with primary antibody for 2 h at room temperature, washed with TBST three times and incubated with secondary antibodies for an additional 1 h at room temperature. DNA was stained with 4,6-diamidino-2-phenylindole for 2–3 min. Coverslips were mounted using ProLong antifade (Sigma). Images were acquired on a DeltaVision microscope (GE Healthcare, Buckinghamshire, UK) with a 60× objective lens, NA=1.42, with optical sections acquired 0.2-0.3 μm apart in the *z*-axis, and were shown as maximal intensity projections. One exposure setting is used within each experiment. Images shown in the same panel have been identically scaled.

### Plasmids and recombinant proteins

CENPB-INCENP-mCherry and wt-INCENP-mCherry or EGFP have been described [34]. Mad2-RFP also has been described [50]. MBP-Haspin, MBP-KD-Haspin, EGFP-Haspin, EGFP-Haspin-11A/E and Histone H3 (1–45) were described previously [14]. Haspin-N-EGFP was constructed by cloning 1-350 aa Haspin cDNA sequence into the *Hin*d111/*Eco*R1 sites of pEGFP-N1 (Clontech, Palo Alto, CA, USA). p3xFlag-AurA/B was constructed by cloning full-length Aurora A/B into the *Bgl*11/*Kpn*1 sites of p3xFlag-Myc-CMV-24 (Sigma). GST-Haspin-N was constructed by cloning 1-350aa Haspin cDNA sequence into the *Bam*H1/*Xho*l1 sites of pGEX-5X-3 (Amersham Biosciences, Buckinghamshire, UK). pmCherry-Aurora-A-WT was constructed by cloning Aurora-A cDNA sequence into the *Bgl*II/*Kpn*I sites of pmCherry-C3 (Clontech). For pmCherry-NES-Aurora-A, the nuclear export signal (NES), 5′-TTACAATTACCTCCTTTAGAACGTTTAACTTTA-3′, encoding LQLPPLERLTL [35] was fused into the N-terminus of Aurora-A cDNA. For pTripz-AurA-shRNA plasmid, AurA-shRNA targeting the AurA cDNA sequence 5′-TCCCAGCGCATTCCTTTGCAA-3′ was inserted into Inducible TRIPZ Lentiviral shRNA (Thermo Scientific, Hudson, NH, USA) according to the technical manual. For shRNA resistance constructs of EGFP-WT/KD/NES-AurA, six sites were mutated in AurA-shRNA targeted sequence without changing the amino acid.

*E. coli* RosettaTM2 (DE3) pLysS cells (TransGen Biotech, Beijing, China) were used to express GST-tag recombinant proteins. A freshly transformed colony was used to initiate a small volume liquid culture in LB medium with 100 μg ml^−1^ ampicillin. This culture was used to inoculate a large volume of the same medium and grown until an absorbance at 600 nm of 0.5 was reached. Protein expression was induced by adding 0.5 mM isopropyl thiogalactoside and growth with shaking at 16 °C for 20 h. Affinity column chromatography was carried out using amylose resin following the manufacturer’s instructions (TransGen Biotech, Beijing, China). The fusion protein was eluted in 10mM GSH in 50 mM Tris pH 8.0. MBP-tag proteins were expressed as described previously [14]. The purity and yield of intact fusion protein were determined by SDS-PAGE and Coomassie Blue staining, in comparison with known quantities of protein marker (ProteinRuler II, TransGen Biotech).

### In vitro kinase reactions and mass spectrometry

*In vitro* phosphorylation of GST-Haspin-N was conducted in 10 μl 2×kinase buffer (50 mM HEPES, 100 mM NaCl, 10 mM MgSO_4_, 2 mM DTT, 4 mM EDTA, pH 7.2) and incubated for 30 min at 30 °C with 1 μg substrate, 130 ng human His-Aurora A (Life Technologies Corporation, Carlsbad, CA, USA), with 50 μM ATP and 5 μCi [^32^P]-ATP. Incorporation of [32] P was visualized by SDS-PAGE and autoradiography. For mass spectrometry and GST-pull-down, the sample was prepared through in vitro kinase reactions except that the 5 μCi [^32^P]-ATP was not added. Mass spectrometry was performed by Shanghai Applied Protein Technology Co. Ltd (Shanghai, China). Phosphorylation of GST-H3(1–45) by MBP-Haspin or MBP-KD-Haspin with or without GST-AurA or GST-KD-AurA for H3T3ph analysis was carried out similarly, but in the presence of 100 mM ATP.

### Acid extraction of histones from HeLa cells

HeLa cells were synchronized by double thymidine (2 mM, Sigma). After releasing, 0.5 μM Noc was added and, 6 h later, the indicated inhibitors were added for 2 h. Mitotic cells were collected by shake-off and the attachment cells were defined as G2-phase cells. Cells were lysed using Hcl-lysis buffer (10 mM HEPES, pH 7.9, 1.5 mM MgCl_2_, 10 mM KCl, 0.5 mM DTT, 1.5 mM PMSF) according to the H3T3ph antibody’s manual (Millipore). Briefly, hydrochloric acid was added to the lysis buffer to a final concentration of 0.2 M (0.2 N) before use. Then, the cell pellet was suspended in 5-10 volumes of lysis buffer and incubated on ice for 30 min. Finally, the supernatant was collected after centrifugation (11 000 *g*, 1 min).

### Immunoprecipitation, GST pull-down assays and immunoblotting

For co-immunoprecipitation (co-IP) of protein complexes, HEK293T cells co-transfected with Flag-tagged and GFP-tagged protein were lysed in co-IP buffer (50 mM Tris HCl, pH 7.4, with 250 mM NaCl, 1 mM EDTA, 50 mM NaF and 0.5% Triton X-100, together with protease inhibitors). The Flag-tagged proteins were precipitated using anti-Flag M2 resin following the manufacturer’s instructions (Sigma). For GST pull-down assays, *E. coli* were lysed in lysis buffer (10 mM Tris pH 7.4, 150 mM NaCl, 0.25% NP-40, 10 mM EDTA and add DTT to 5 mM, PMSF to 5 mM and protease inhibitors prior to use) by sonication. GST-tagged proteins precipitated by affinity column chromatography were carried out using amylose resin following the manufacturer’s instructions (TransGen Biotech). Purified MBP-Haspin protein was incubated with amylose resin containing GST-tagged protein for 2 h at 4 °C and eluted using 10 mM GSH in 50 mM Tris pH 8.0. For GST-tagged protein precipitation, Flag-tagged proteins expressed in HEK293T cells were incubated with anti-Flag M2 resin, washed with TBS and incubated with purified GST-Haspin-N protein, which had been phosphorylated by rhAurA or not as described above for 2 h at 4 °C, washed with TBS, and bound proteins were eluted using Flag peptide.

For immunoblotting, proteins were resolved by SDS-PAGE and transferred onto polyvinylidene difluoride (PVDF) membranes (Millipore). Immunoblots were developed in Western Lightning Chemiluminescence Reagent Plus (Advansta, Menlo Park, CA, USA). When necessary, blots were stripped and reblotted with other antibodies.

### Live-cell imaging

For live-cell imaging, cells on coverslips were mounted in Rose chambers and maintained at 37 °C in phenol-free L-15 medium (Invitrogen) with 10% fetal bovine serum. Time-lapse images were acquired at 1-5 min intervals with a 100×1.4 NA PlanApo objective lens mounted on an Eclipse Ti microscope (Nikon, Tokyo, Japan). Z-stacks were collected at 1-μm steps with a sCMOS camera (C11440, HAMAMATSU, Hamamatsu City, Japan). Integrated fluorescence intensities were measured as previously described [49]. Images were deconvolved using SoftWorx software (Applied Precision, Issaquah, WA, USA).

### Checkpoint recovery assay

Hela cells transiently transfected with Mad2-RFP were incubated for 30 min with MG132 (10 μM, Selleck Chemicals) to arrest cells at metaphase. Inhibitors were added 30 min before addition of Noc (3.3 μM, Invitrogen). Time-lapse images were taken every 1 min for 30 min after Noc was added. Images were deconvolved and were shown as maximal intensity projections using SoftWorx software (Applied Precision).

### Statistical analysis

Quantification of immunofluorescence intensities was carried out using ImageJ (NIH, Bethesda, MD, USA) as described previously [49]. For Aurora B, Mad2, BubR1, Bub1, and H2AT120ph, because the distance between the sister kinetochores or centromeres was too close to measure separately, sister kinetochore or centromere pairs in the same section were analyzed together. In addition, the intensities were normalized to the ACA levels of the corresponding centromere pairs. For H3T3 or H3S10 intensity, images were projected as maximal intensity projections and integrated intensities of the full nucleus were measured by ImageJ. Data were from three or more independent experiments. Statistical analyses were carried out by GraphPad Prism 5 software (La Jolla, CA, USA).
