# Supplementary material for: Aurora-A promotes the establishment of spindle assembly checkpoint by priming the Haspin-Aurora-B feedback loop in late G2 phase
Source: Cell Discov. 2017 Jan 10;3:16049–. doi: 10.1038/celldisc.2016.49 (PMC5223110; doi:10.1038/celldisc.2016.49)
Supplement: Supplementary Information [file celldisc201649-s1.pdf]

Figure S1

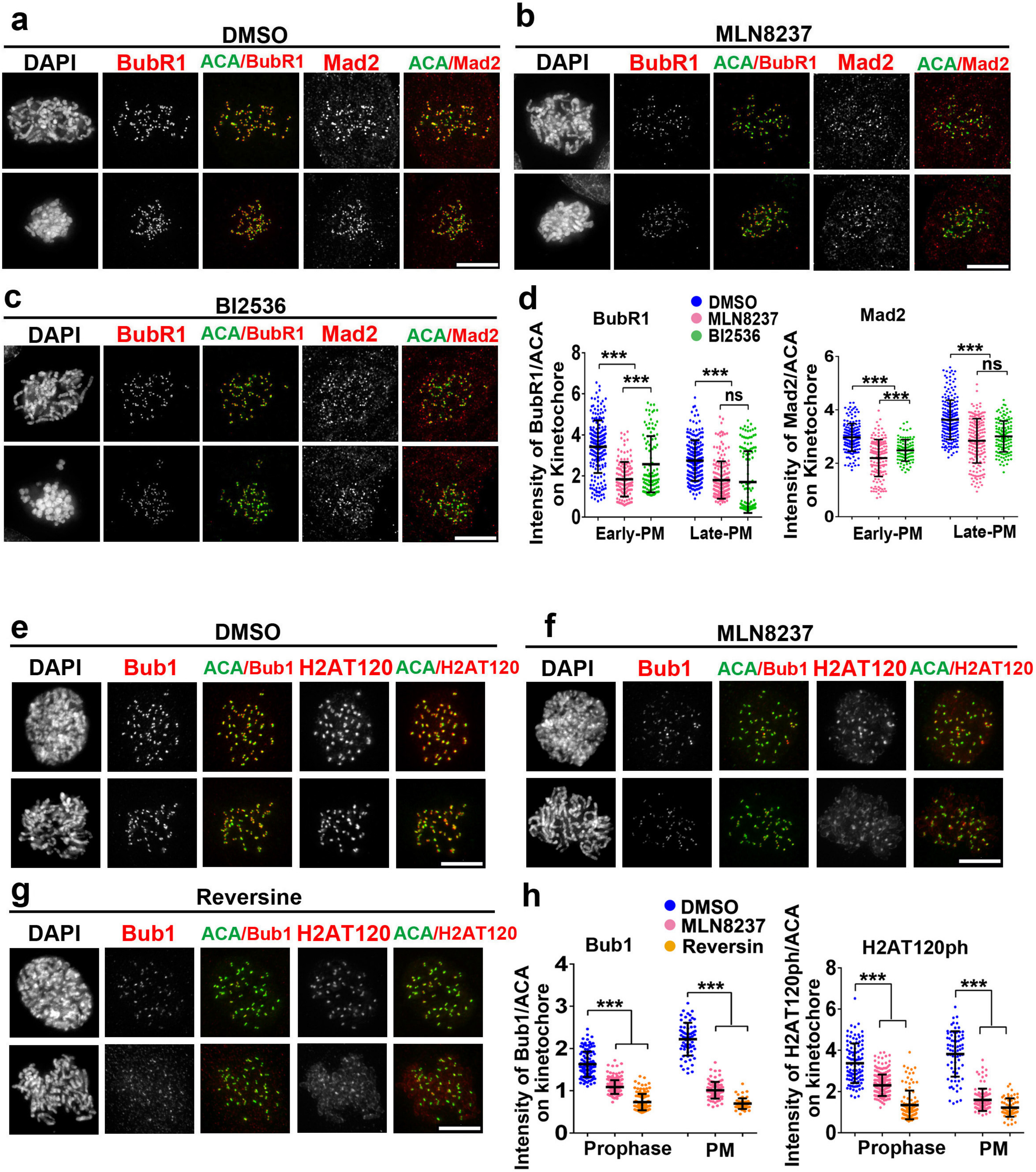
a
DMSO
DAPI
BubR1
ACA/BubR1
Mad2
ACA/Mad2
b
MLN8237
c
BI2536
d
BubR1
Mad2
DMSO
MLN8237
BI2536
Intensity of BubR1/ACA on Kinetochore
Intensity of Mad2/ACA on Kinetochore
***
ns
Early-PM
Late-PM
e
DMSO
DAPI
Bub1
ACA/Bub1
H2AT120
ACA/H2AT120
f
MLN8237
g
Reversine
h
DMSO
MLN8237
Reversin
Bub1
H2AT120ph
Intensity of Bub1/ACA on kinetochore
Intensity of H2AT120ph/ACA on kinetochore
Prophase
PM

Figure S2

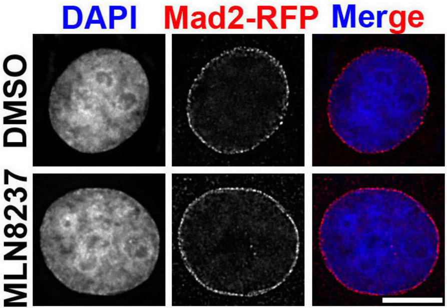

Figure S3

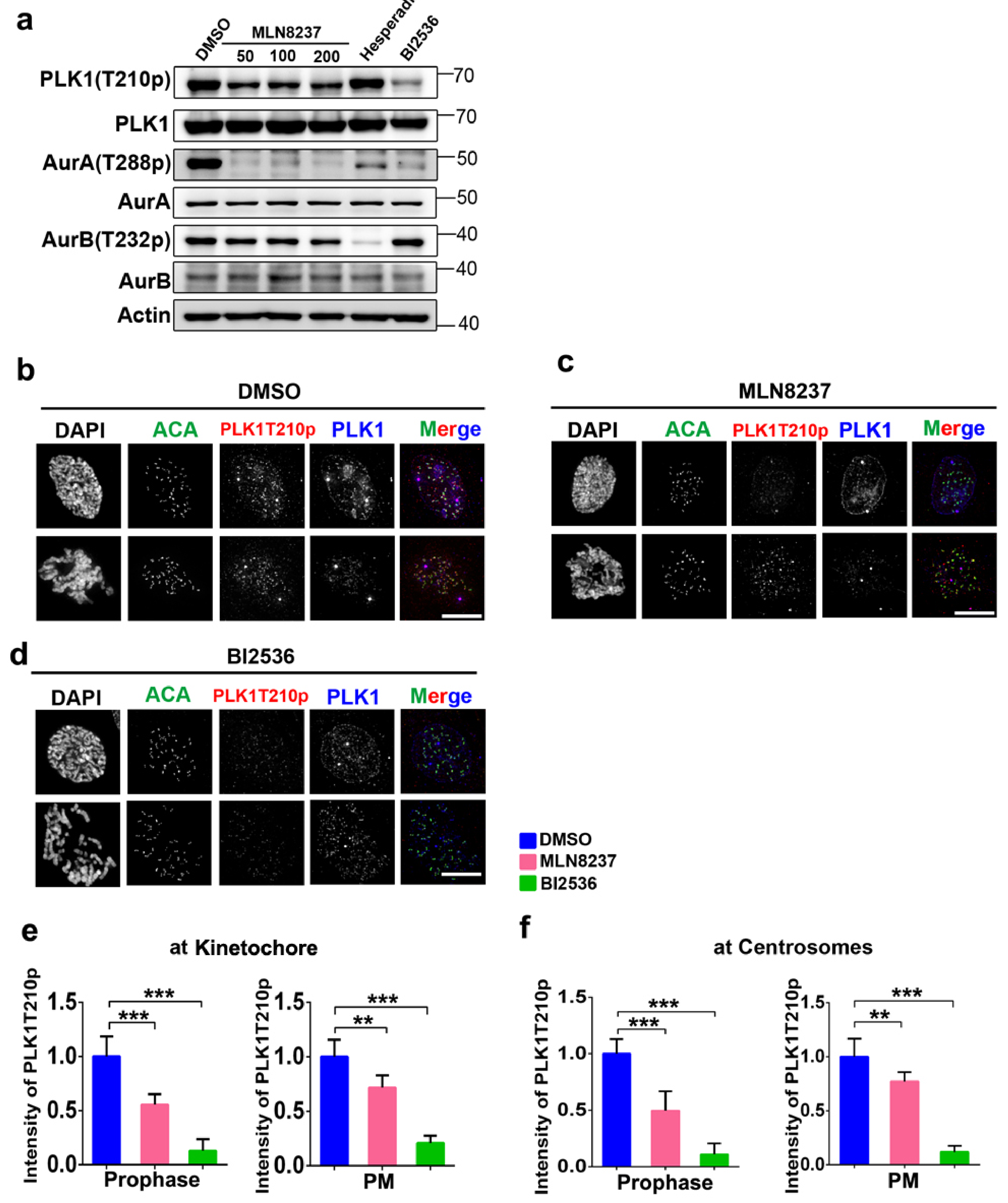
a
DMSO
MLN8237
50
100
200
Hesperadin
BI2536
PLK1(T210p)
PLK1
AurA(T288p)
AurA
AurB(T232p)
AurB
Actin
70
70
50
50
40
40
40
b
DMSO
DAPI
ACA
PLK1T210p
PLK1
Merge
c
MLN8237
DAPI
ACA
PLK1T210p
PLK1
Merge
d
BI2536
DAPI
ACA
PLK1T210p
PLK1
Merge
DMSO
MLN8237
BI2536
e
at Kinetochore
Intensity of PLK1T210p
1.5
1.0
0.5
0.0
Prophase
PM
***
**
f
at Centrosomes

Figure S4

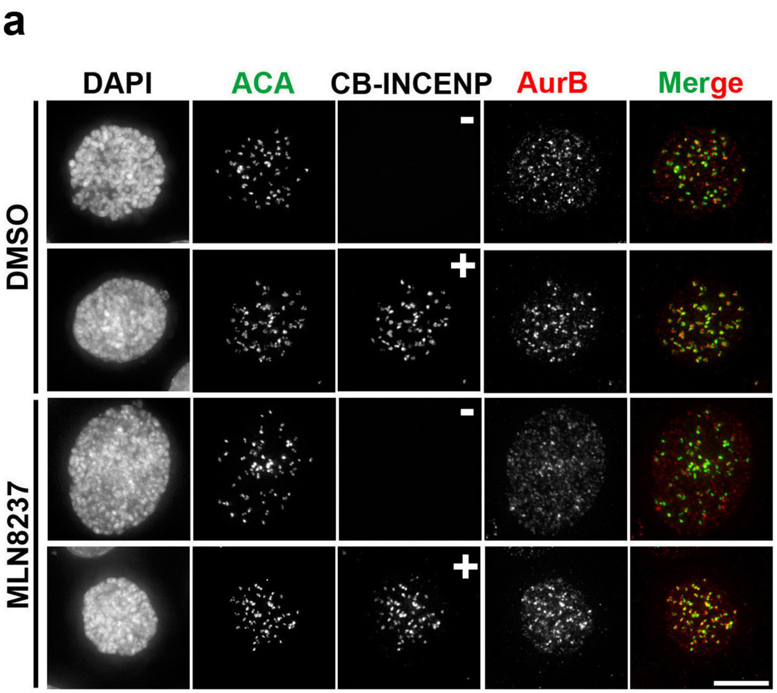

a
DAPI
ACA
CB-INCENP
AurB
Merge
DMSO
MLN8237
-
+
-
+

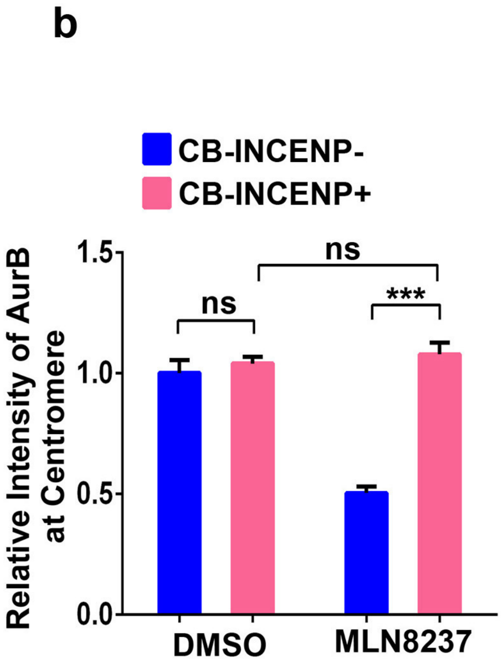

b
CB-INCENP-
CB-INCENP+
Relative Intensity of AurB at Centromere
1.5
1.0
0.5
0.0
ns
ns
***
DMSO
MLN8237

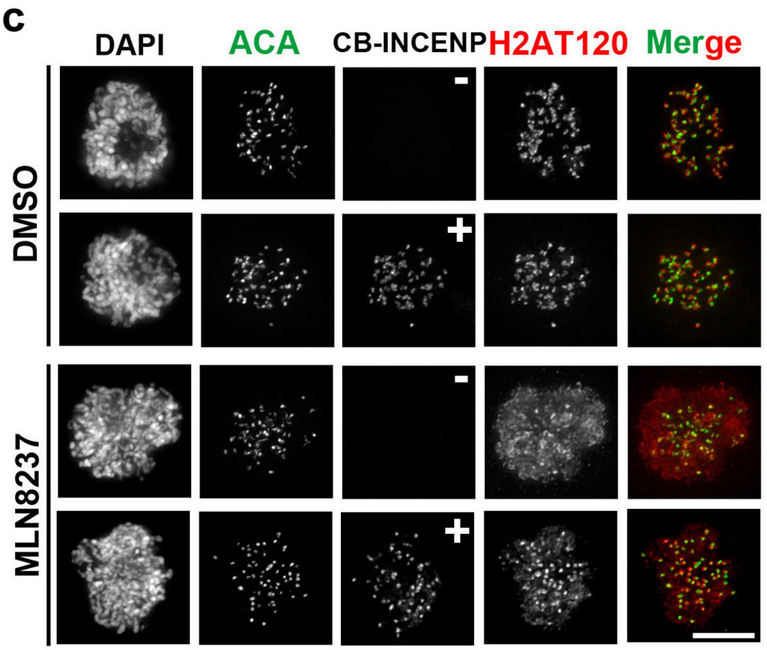

c
DAPI
ACA
CB-INCENP
H2AT120
Merge
DMSO
MLN8237
-
+
-
+

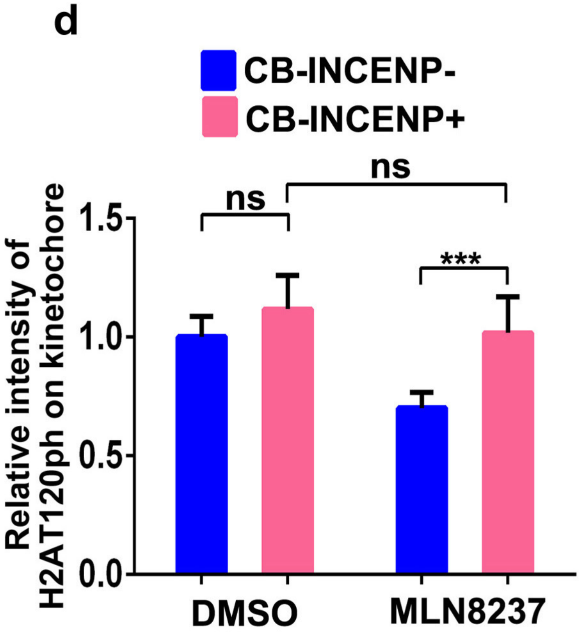

d
CB-INCENP-
CB-INCENP+
Relative intensity of H2AT120ph on kinetochore
1.5
1.0
0.5
0.0
ns
ns
***
DMSO
MLN8237

Figure S5

a b

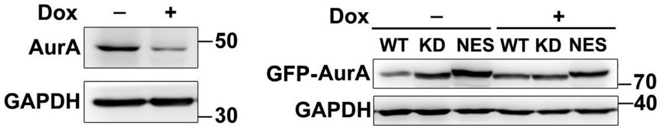

c

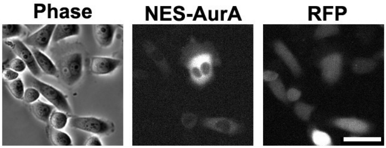

	EGFP-C1	WT-AurA	KD-AurA	NES-AurA
Muti-nuclear cells	0	0	5	9
Total cell number	153	139	138	137

d e

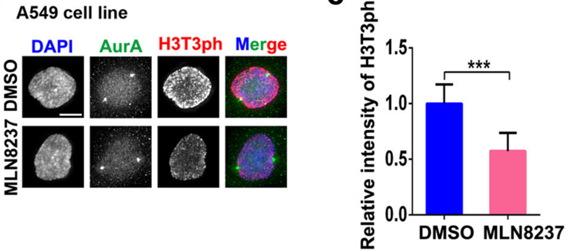

Figure S6

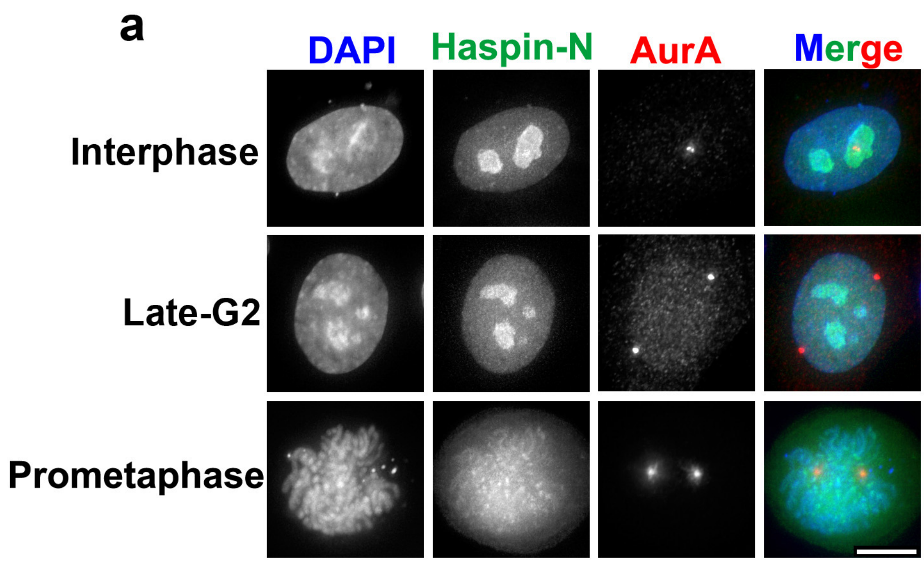

b

Sequence surrounding site	Phosphorylation sites	Frequency
K. DRPS#LTVTPK. R	S93	4
K. DRPS#LTVTPK. R	S93	
K. DRPS#LTVTPK. R	S93	
K. DRPS#LTVTPK. R	S93	
R. ARPS#LTVTPR. R	S108	6
R. ARPS#LTVTPR. R	S108	
R. ARPS#LTVTPR. R	S108	
R. ARPS#LTVTPR. R	S108	
R. ARPS#LTVTPR. R	S108	
R. ARPS#LTVTPR. R	S108	
R. LPPFPSRDS#GR. L	S143	4
R. LPPFPSRDS#GR. L	S143	
R. LPPFPSRDS#GR. L	S143	
R. LPPFPSRDS#GR. L	S143	
R. LPPFPSRDS#GR. L	S143	
R. DSGRLS#PDLSVCGQPR. D	S147	3
R. DSGRLS#PDLSVCGQPR. D	S147	
R. DSGRLS#PDLSVCGQPR. D	S147	
R. AS#LPCSQEEATGGAK. D	S216	2
R. AS#LPCSQEEATGGAK. D	S216	

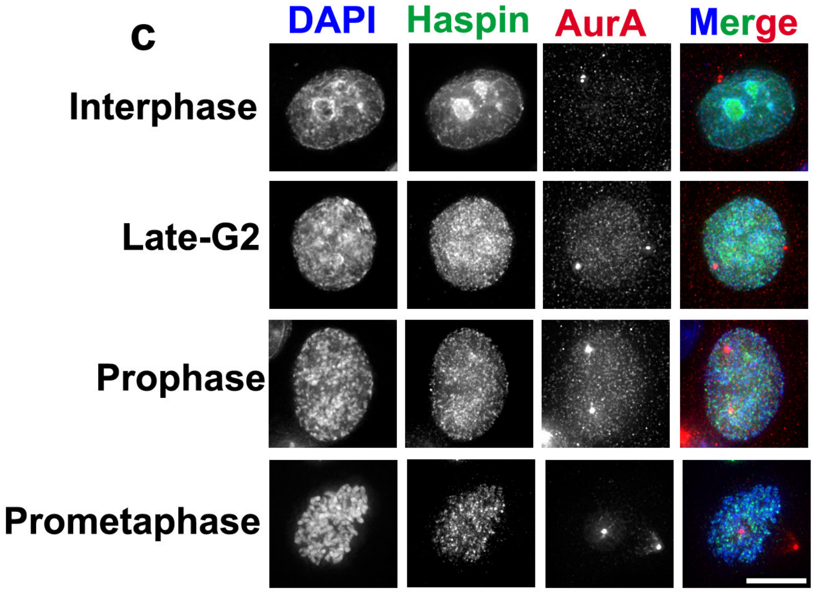

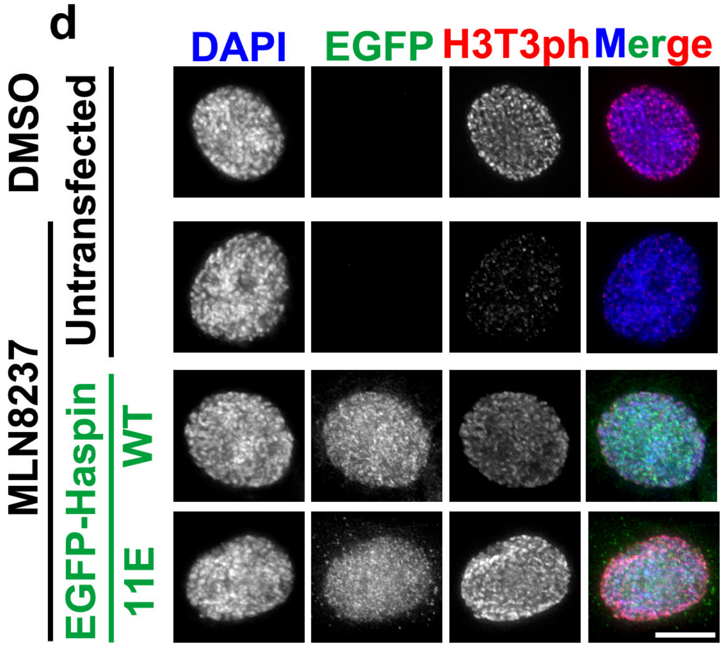

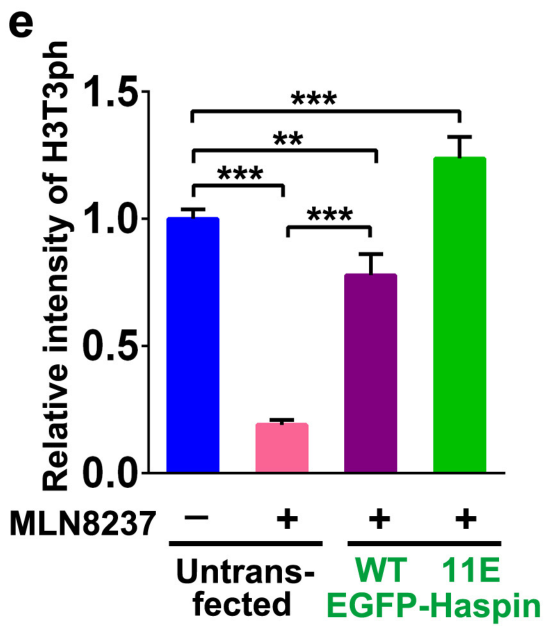

Supplementary Figure Legends

Supplementary Figure S1. Aurora-A is essential for SAC establishment in early mitosis. (**a-c**) RPE cells were treated with DMSO (**a**), MLN8237 (**b**) or BI2536 (**c**) for Two hours. Mad2, BubR1 and ACA were simultaneously examined by immunofluorescence in the same cell. (**d**) The relative intensity of Mad2 and BubR1 in early and late prometaphase was measured and quantified. 200 kinetochores from 60 cells were included. Cells analyzed were from at least three independent experiments. PM: prometaphase. (**e-g**) RPE cells were treated with DMSO (**e**), MLN8237 (**f**) or Mps1 inhibitor: Reversine (**g**) for Two hours. Bub1, H2AT120ph and ACA were examined by immunofluorescence. (**h**) The relative intensity of Bub1 and H2AT120ph in prophase and prometaphase cells was measured and quantified. 200 kinetochores from 60 cells were included. Cells were analyzed from three independent experiments. PM: prometaphase. Scale bar: 10 μm.

Supplementary Figure S2. Aurora-A is not required for the recruitment of Mad2 to nuclear envelope. Hela cells were transfected with Mad2-RFP and treated with DMSO or MLN8237. Mad2 fluorescence at nuclear pore complexes were monitored.

Supplementary Figure S3. Aurora-A inhibition by MLN8237 slightly reduced Plk1 kinase activity, but did not affect Aurora-B kinase activity. (a) HeLa cells were arrested in mitosis with Noc for 18h and were treated with MLN8237 (50nM, 100nM and 200nM), Hesperadin (100nM) and BI2536 (100nM) for 2h. Cells were harvested by shake-off and subjected for immunostaining to assess the activities of kinases with antibodies specific phosphorylated proteins. (**b-d**)The level of active Plk1 was examine by immune-staining in cells treated with DMSO **(b)**, MLN8237 (50nM) **(c)** and BI2536 **(d)** for 2h. (**e, f**)The relative intensity of Plk1T210p at kinetochores **(e)** and centrosomes **(f)** was quantified. PM: prometaphase. Cells (n=60) analyzed were from three independent experiments. Values represent as mean ± SEM. ***P<0.001 (Student's t-test), Scale bar: 10 μm.

Supplementary Figure S4. CB-INCENP tethers Aurora-B at centromeres in early mitosis and rescues kinetochore localization of H2AT120 when Aurora-A was inhibited. (**a**) CENP-B-INCENP-mCherry (CB-INCENP) fusion protein was transfected into Hela cells. The cells were then treated with DMSO or MLN8237 for Two hours before fixation. Aurora-B and ACA were labeled. (**b**) The intensity of Aurora-B at centromeres was measured. Cells (n=40) analyzed were from four independent experiments. (**c**) CB-INCENP was transfected into Hela cells. The cells were then treated with DMSO or MLN8237 for Two hours before fixation. H2AT120ph and ACA were labeled. (**d**) The intensity of H2AT120ph was measured. Cells (n=40) analyzed were from four independent experiments. Values represent as mean ± SEM. ***P<0.001 (Student's t-test), ns: not significant. Scale bar: 10 μm.

Supplementary Figure S5. NES-Aurora-A expression with endogenous Aurora-A knocked-down induces bi-nucleated cells. (a) Endogenous Aurora-A was knocked-down with a Doxycycline induced shRNA in HeLa cells. **(b)** Wild type (WT), kinase dead (KD) or nuclear export signal tagged (NES) Aurora-A was expressed by shRNA-resistant constructs when shRNA was induced by doxycycline (dox). **(c)** Typical images of bi-nucleated cells in NES-Aurora-A expressed cells and statistics of bi-nucleated cells in Aurora-A constructs transfected cells. RFP was the reporter of induced shRNA. Scale bar: 20 μm. **(d)** Human cancer cell line A549 were treated with 50 nM MLN8237, H3T3ph level was examined by immunostaining in late G2 cells. Scale bar: 10 μm. **(e)** the relative intensity of H3T3ph was quantified (n=90-100), ***P<0.001 (Student's t-test).

Supplementary Figure S6. Aurora-A interacts with and regulates Haspin. (**a**) Co-localization of Haspin-N-GFP and endogenous Aurora-A in G2 phase. (**b**) The phosphorylation sites on GST-Haspin-N were identified using mass spectrum analysis of samples from the in vitro kinase assays. (**c**) Co-localization of Haspin-GFP and endogenous Aurora-A in the nucleus in late G2 phase and prophase. (**d**) Hela cells transfected with wild-type Haspin (WT) and Haspin 11E were treated with MLN8237. H3T3ph level were examined in prophase cells. **(e)** Quantification of H3T3ph in cells

in **c**. Cells (n=60) analyzed were from three independent experiments. Values represent as mean ± SEM. ***P<0.001, **P<0.01 (Student's t-test). Scale bar: 10 μm.
